# Supplementary material for: The complex of ferric-enterobactin with its transporter from Pseudomonas aeruginosa suggests a two-site model
Source: Nat Commun. 2019 Aug 14;10:3673. doi: 10.1038/s41467-019-11508-y (PMC6694100; doi:10.1038/s41467-019-11508-y)
Supplement: Supplementary file 1 — Supplementary Information [file 41467_2019_11508_MOESM1_ESM.pdf]

Supplementary Information

The complex of ferric-enterobactin with its transporter from *P. aeruginosa* suggests a two-site model

Lucile Moynié[1,2,3^], Stefan Milenkovic[4^], Gaëtan L. A. Mislin[6,7^], Véronique Gasser[6,7], Giuliano Malloci[4], Etienne Baco[6,7] Rory P. McCaughan[8], Malcolm G. P. Page[9], Isabelle J. Schalk[5,6,*], Matteo Ceccarelli[4,5,*], James H Naismith[1,2,3*]

Affiliations:

1 Division of Structural Biology, Wellcome Trust Centre of Human Genomics, 7 Roosevelt Drive, Oxford, OX3 7BN
2 The Research Complex at Harwell, Harwell Campus, Oxfordshire, UK OX11 0FA
3 The Rosalind Franklin Institute, Didcot OX11 0FA
4 Department of Physics, University of Cagliari, Cittadella Universitaria, SP Monserrato-Sestu Km 0.700, Monserrato, 09042-Italy
5 Istituto Officina dei Materiali-CNR, Cittadella Universitaria, 09042 Monserrato, Italy
6 Université de Strasbourg, UMR7242, ESBS, Bld Sébastien Brant, F-67413 Illkirch, Strasbourg, France
7 CNRS, UMR7242, ESBS, Bld Sébastien Brant, F-67413 Illkirch, Strasbourg, France
8 BSRC, The University of St Andrews, KY16 9ST
9 Department of Life Sciences & Chemistry, Campus Ring 1, 28759 Bremen Germany

^ These authors contributed equally to this work: Lucile Moynié, Stefan Milenkovic, Gaëtan L. A. Mislin.

* Correspondence and requests for materials should be addressed to I.J.S. (email: schalk@unistra.fr), M.C. (email: matteo.ceccarelli@dsf.unica.it) or J.H.N. (email: naismith@strubi.ox.ac.uk)

Keywords: Enterobactin, structural biology, TBD uptake, iron uptake

Author contributions

LM performed all the crystallography, biophysics, analysed data and wrote paper

GM performed docking calculations and force field of enterobactin-iron complex

SM performed molecular dynamics simulations, analysed data and wrote paper

MC directed project, analysed data and wrote paper

MGPP secured funding and directed project

RPM performed mutant proteins purification and crystallization

JHN directed project, analysed data and wrote paper

VG performed ^{55}Fe uptake assay and construct some of the strains

EB performed azotochelin and protochelin synthesis and purification

GLAM designed the syntheses and analysed chemical data

IJS directed project, analysed data and wrote paper

Competing interests:

The authors declare no competing interests.

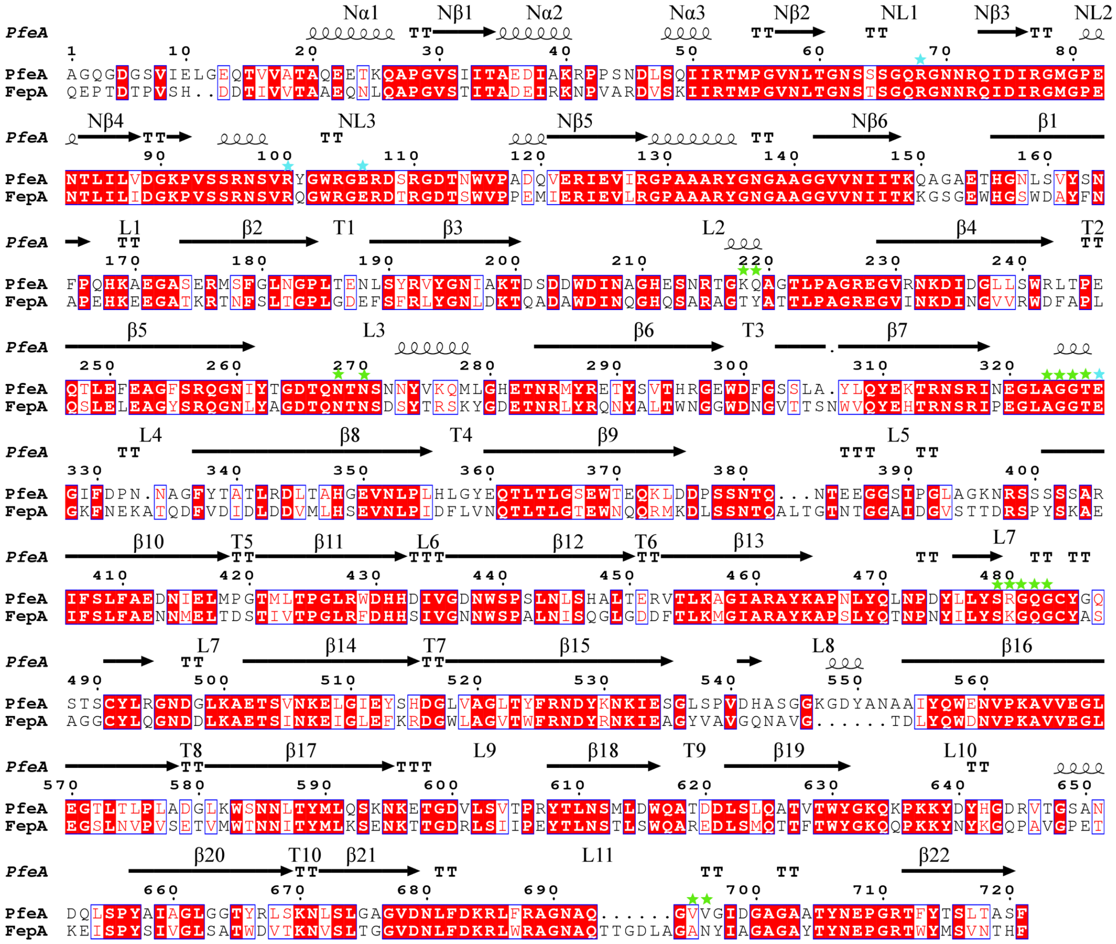

Supplementary Figure 1. PfeA secondary structure. Secondary structure elements of PfeA are illustrated above the sequence. Residues forming the binding site are highlighted with a green star and the ones involved in H-bonds transmission system with a cyan star. Nα and Nβ refer to the plug domain elements. Similarities between the two sequences are rendered with a blue frame. Strictly conserved residues are in white characters on a red background and residues similar are in red characters on a white background. Picture was drawn using ESPript[1].

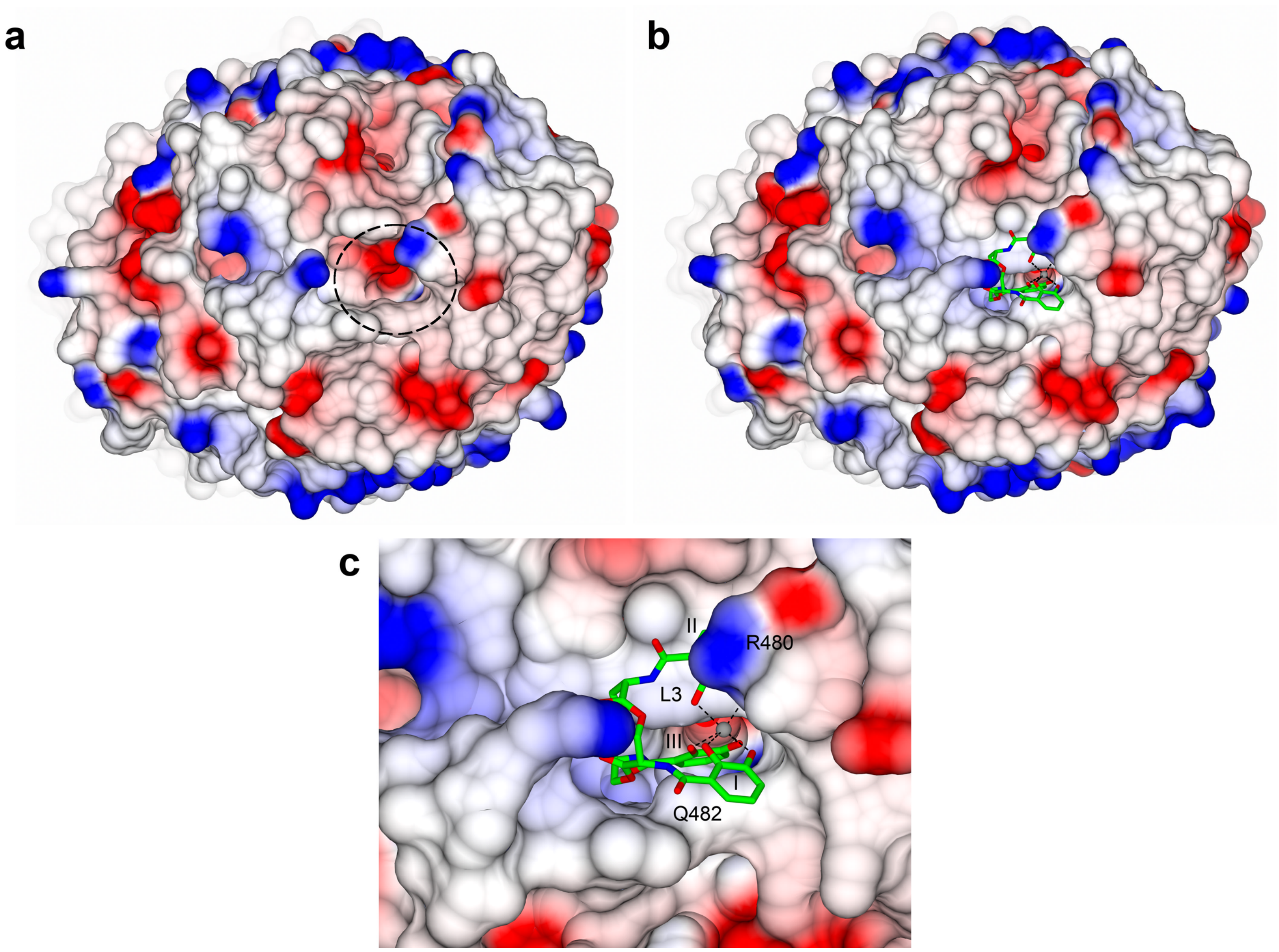

Supplementary Figure 2. View from the extracellular side of PfeA apo and complex structure. The surface of PfeA is colored by electrostatic charges calculated in CCP4MG (red for negative potential, white for neutral and blue for positive). Position of the entrance of the polar cavity has been highlighted with a black circle in the apo structure (**a**). In the complex structure (**b** and **c**), enterobactin is shown as sticks with the carbon in green. A zoom of the enterobactin binding site is represented in **c**.

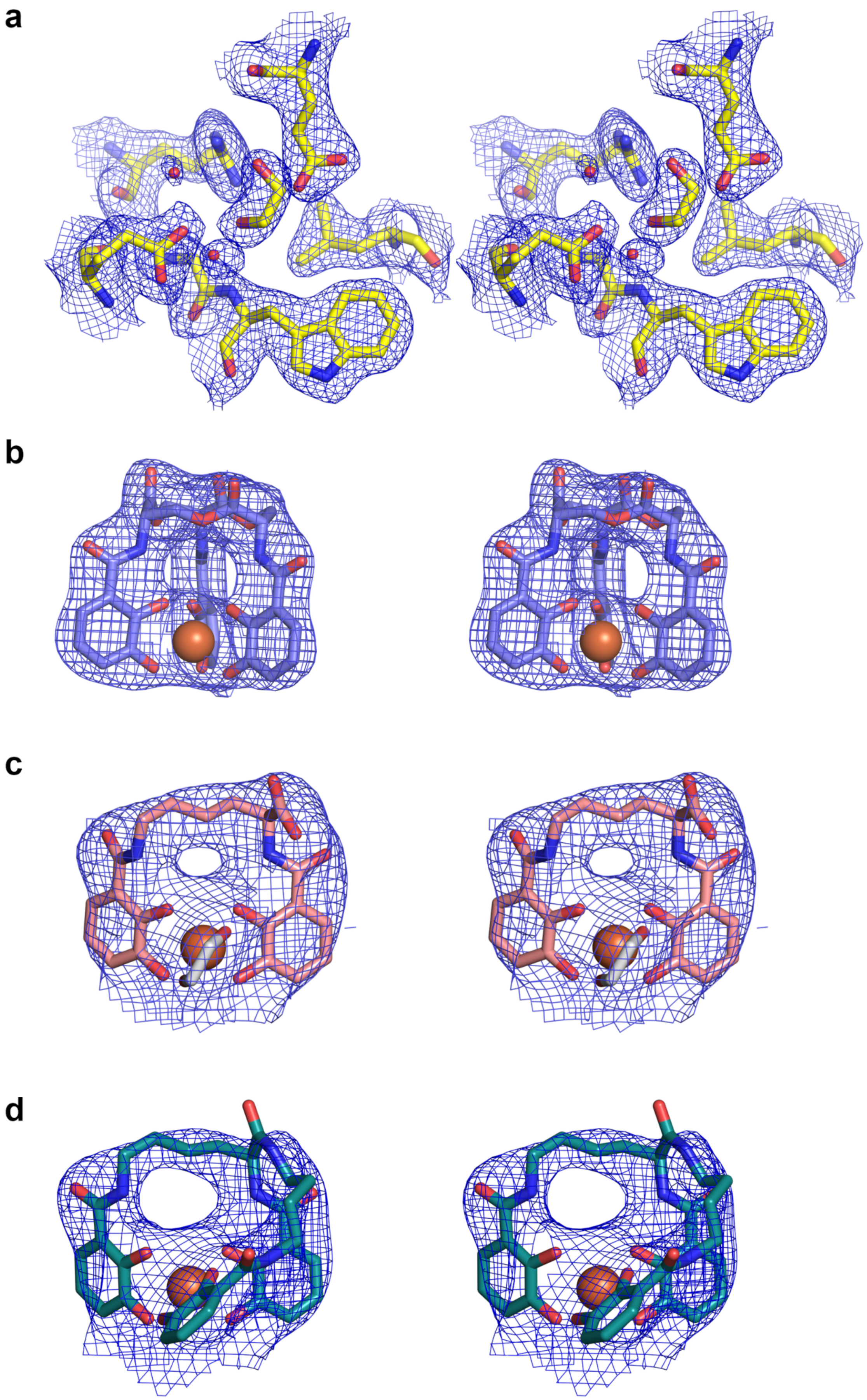
a
b
c
d

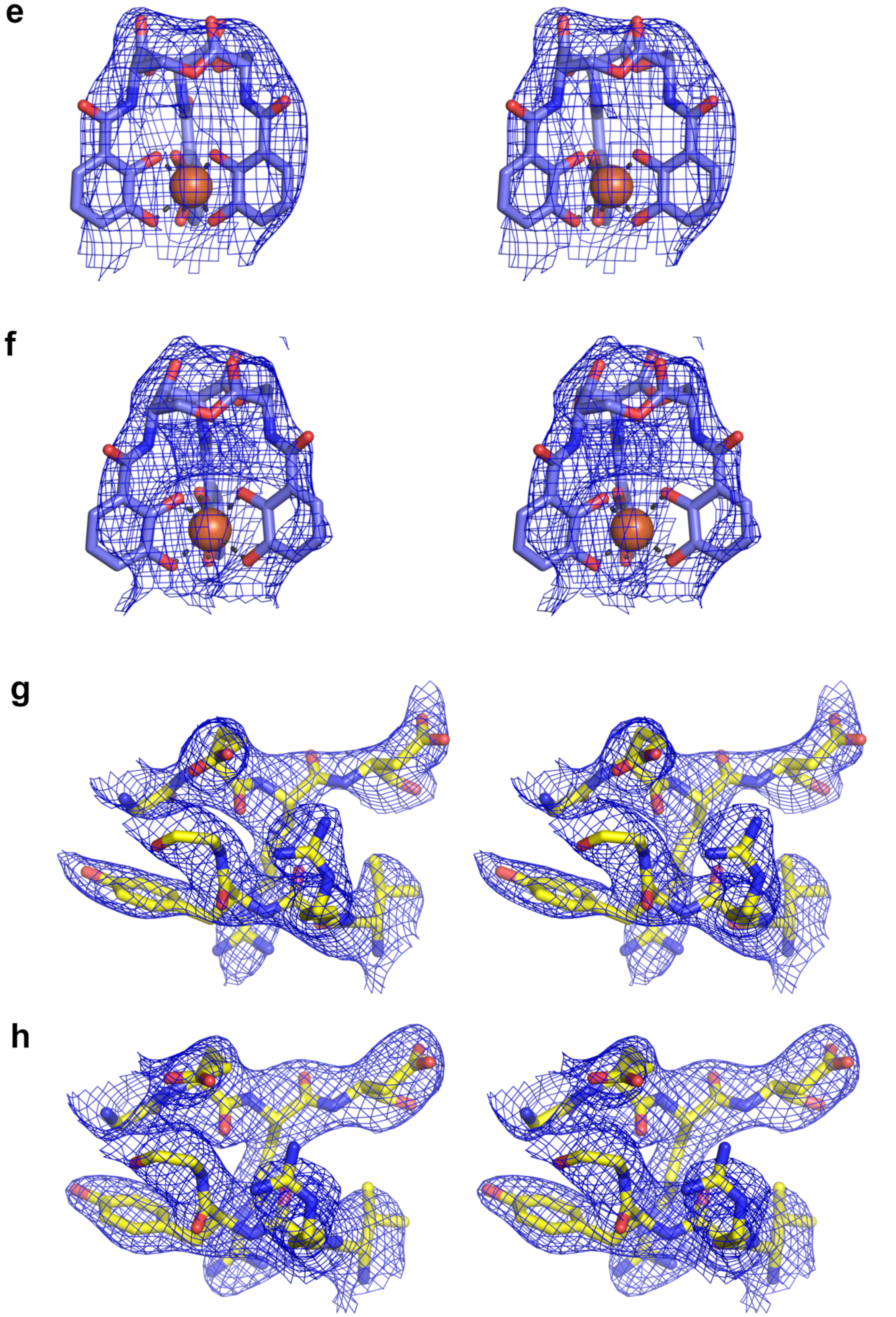

Supplementary Figure 3. Stereo views of a portion of the final $2F_O$-F_C electron density maps. Maps are contoured at 1 σ. Proteins are shown as sticks with carbon atoms colored in yellow, nitrogen in blue, oxygen in red. Carbon atoms of enterobactin, azotochelin and protochelin are colored in blue, salmon and green respectively and Fe^{3+} are represented as an orange sphere. **a** Map around the ethylene glycol bound in the extracellular polar void of PfeA structure. **b** Map around the Fe^{3+}-enterobactin present in the PfeA complex structure. **c** Map

around Fe^{3+}-azotochelin present in the PfeA complex structure. **d** Map around Fe^{3+}-protochelin present in the PfeA complex structure. **e** Map around Fe^{3+}-enterobactin of PfeAR480A complex structure. **f** Map around Fe^{3+}-enterobactin of the PfeAQ482A complex structure. **g** Portion of the plug domain loops NL1 and NL2 of PfeAG324V. **h** Portion of the plug domain loops NL1 and NL2 of PfeAR480A-Q482A.

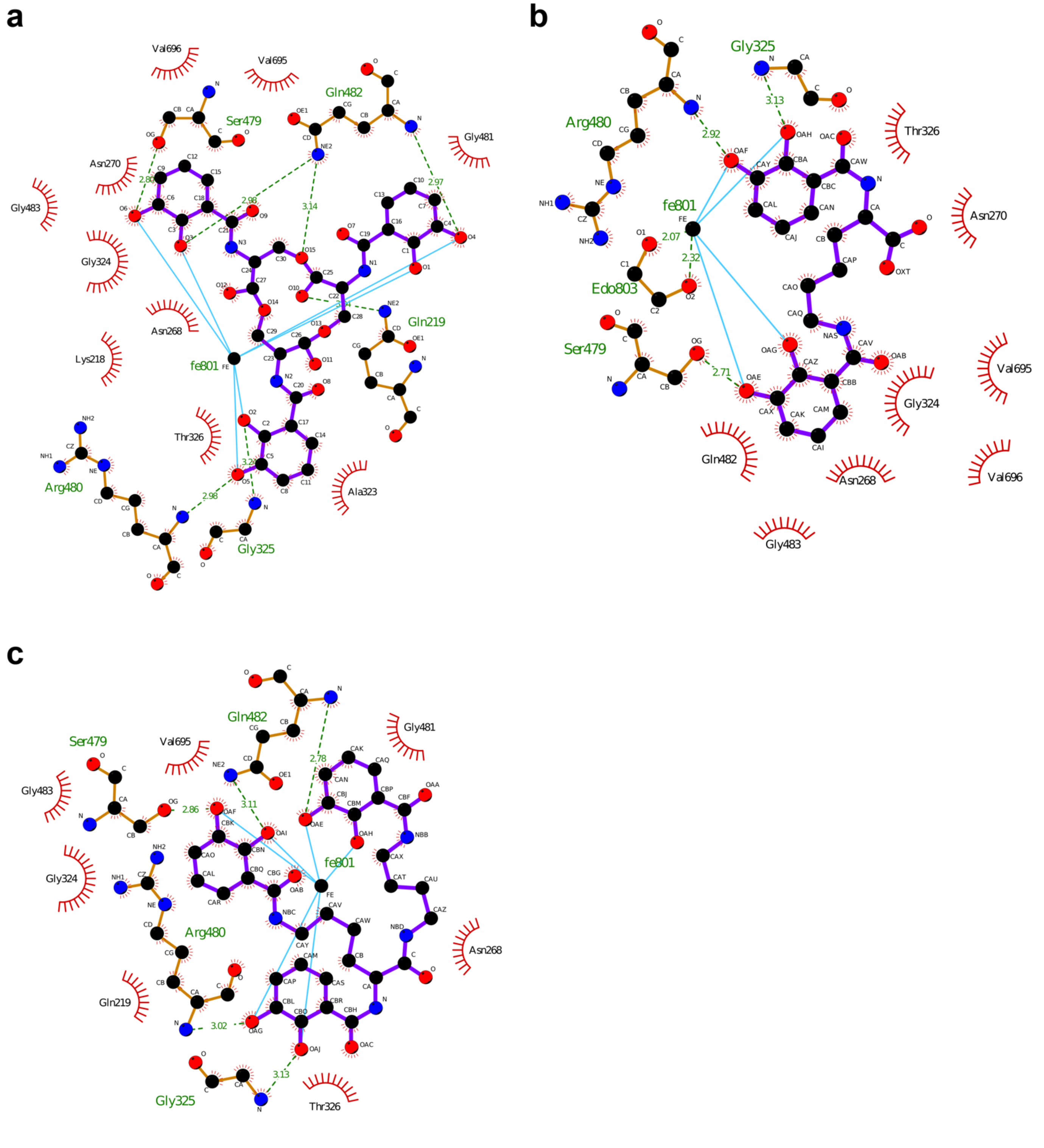

Supplementary Figure 4. Ligplot diagrams illustrating PfeA-siderophore interactions. Fe^{3+}-enterobactin **(a)**, Fe^{3+}-azotochelin (**b**), Fe^{3+}-protochelin (**c**) bound to PfeA. Covalent bonds of the siderophores and protein residues are in purple and brown sticks, respectively. Hydrogen bonds are represented by green dashed lines and hydrophobic contacts are shown as red semi-circles with radiating spokes. Metal interactions are represented by cyan lines. Figure prepared with Ligplot[2].

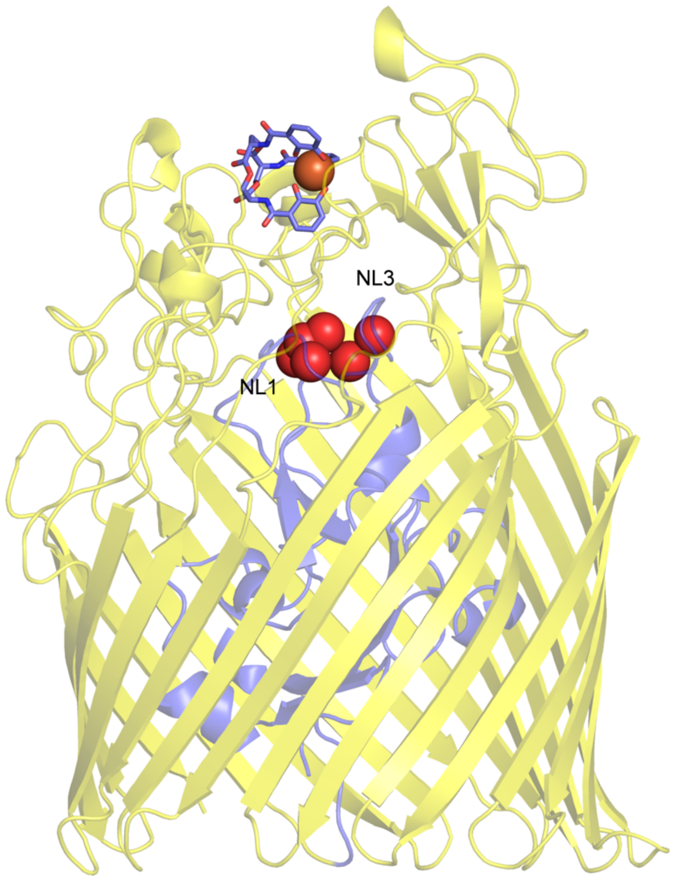

Supplementary Figure 5. Fe^{3+}-enterobactin binds at a different location than other known co-complex siderophores. For clarity, only the iron atoms (red balls) of FptA[3], FpvA[4], FecA[5], FhuA[6], FetA[7] have been represented. PfeA structure is shown in transparency (yellow) and the plug domain is highlighted in blue. Enterobactin is shown as sticks with carbon atoms colored in blue, nitrogen in dark blue and oxygen in red. The Fe^{3+} is represented as an orange sphere.

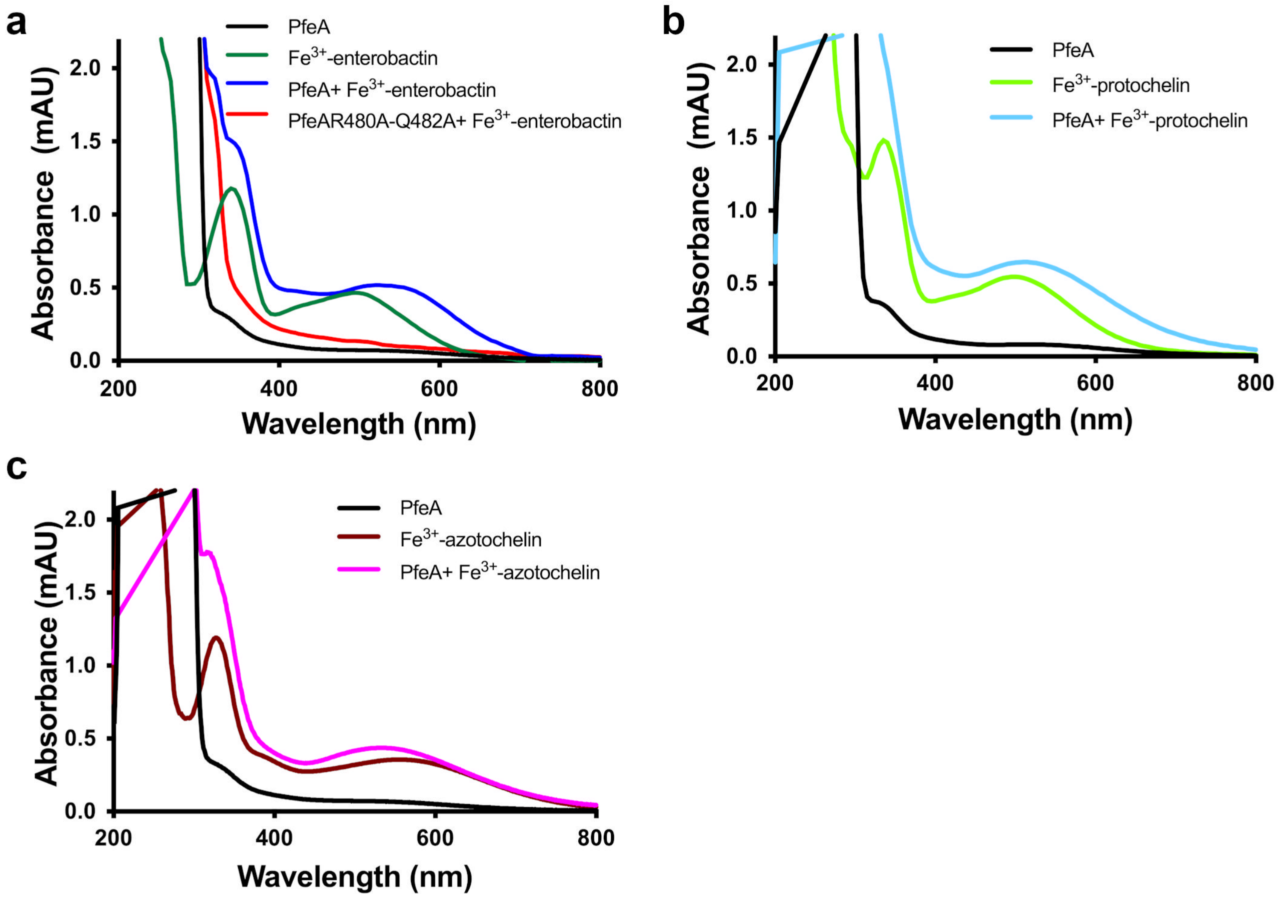

Supplementary Figure 6. PfeA binds Fe^{3+}-enterobactin, Fe^{3+}-protochelin and Fe^{3+}-azotochelin. PfeA proteins were incubated with Fe^{3+}-enterobactin, **(a)** Fe^{3+}-protochelin **(b)** and Fe^{3+}-azotochelin (**c**) before being loaded on a S200 size exclusion column. UV-visible spectra of eluted proteins show a peak of absorption around 550 nm characteristic of the iron-siderophore complex. Unlike the wild-type protein (**a**, blue line), the double mutant R480A-Q482A (**a**, red line) doesn't coelute with Fe^{3+}-enterobactin. Both Fe^{3+}-protochelin and Fe^{3+}-azotochelin coelute with PfeA.

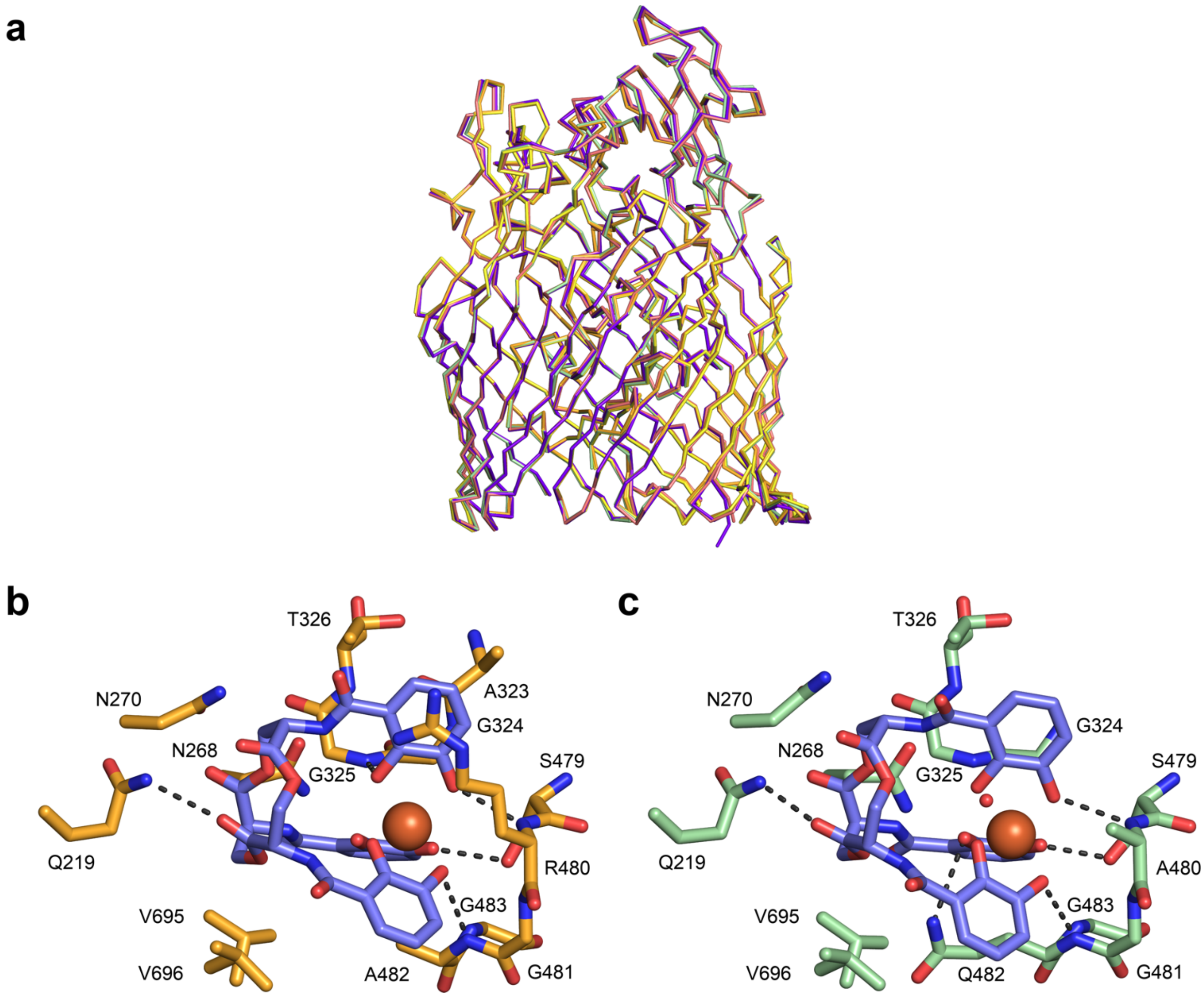

Supplementary Figure 7. **All the mutants have the same conformation as the wild-type.**
a Mutants R480A (green), Q482A (orange), G324V (purple), R480A-Q482A (salmon) superpose with the wild-type with a r.m.s.d. less than 0.24 Å. Fe^{3+}-enterobactin binding site of Q482A (**b**) and R480A (**c**) mutants are the same as the wild-type. Residues within 4.0 Å of the siderophore are displayed and hydrogen bonds are shown as black broken lines. Carbon atoms of PfeAQ482A and PfeAR480A are in orange and green respectively. Enterobactin is shown as sticks with carbon atoms colored in blue, nitrogen in dark blue and oxygen in red, the Fe^{3+} is represented as an orange sphere.

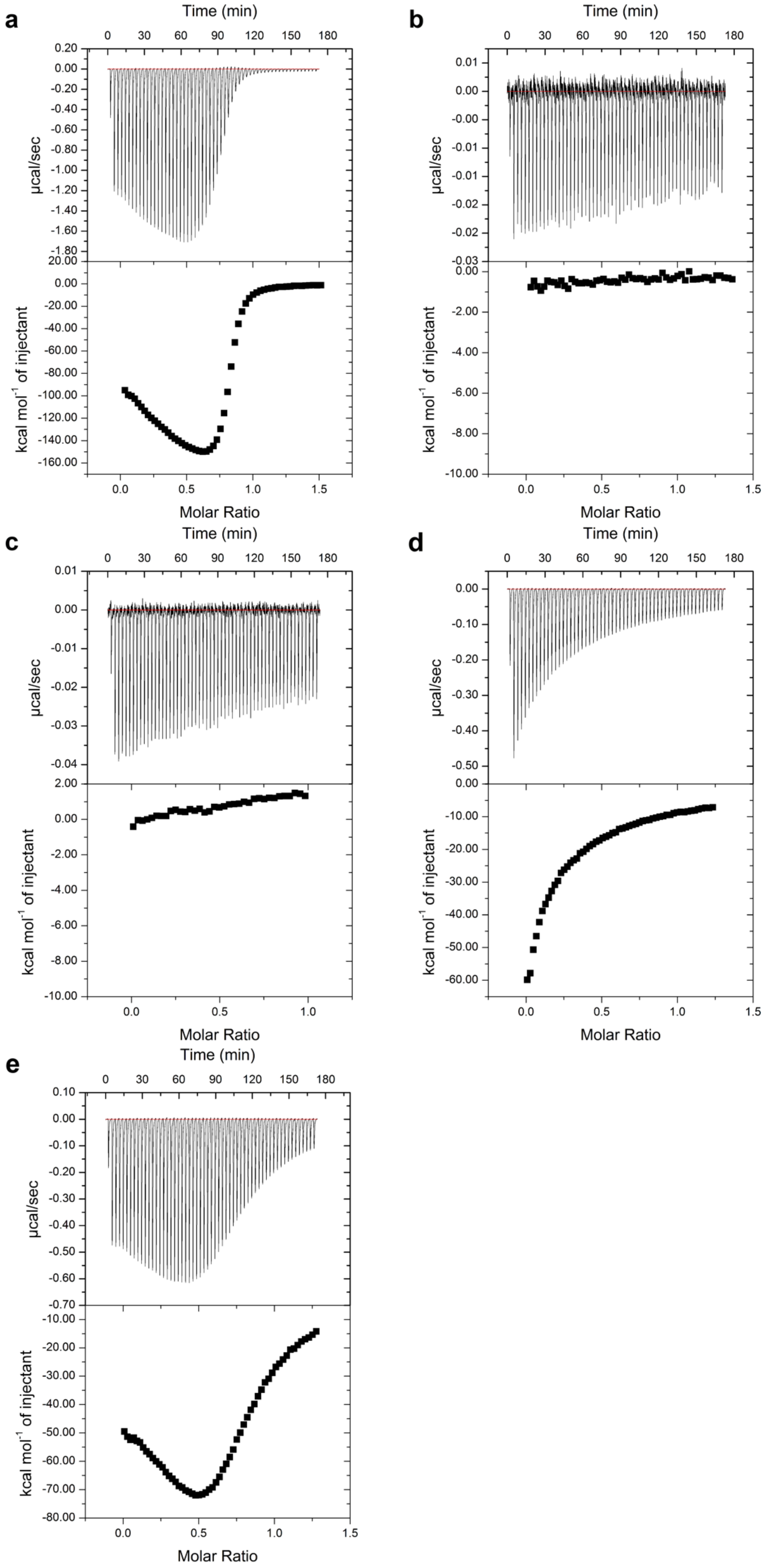
a
b
c
d
e
Time (min)
µcal/sec
kcal mol^{-1} of injectant
Molar Ratio

Supplementary Figure 8. Isothermal calorimetry titrations data. Titrations of Fe^{3+}-enterobactin into PfeA (**a**) and mutants R480A-Q482A (**b**), G324V (**c**), R480A (**d**) and Q482A (**e**) are shown. Top panels correspond to raw titration data and the bottom panels to the isotherms after the control subtraction using Origin software.

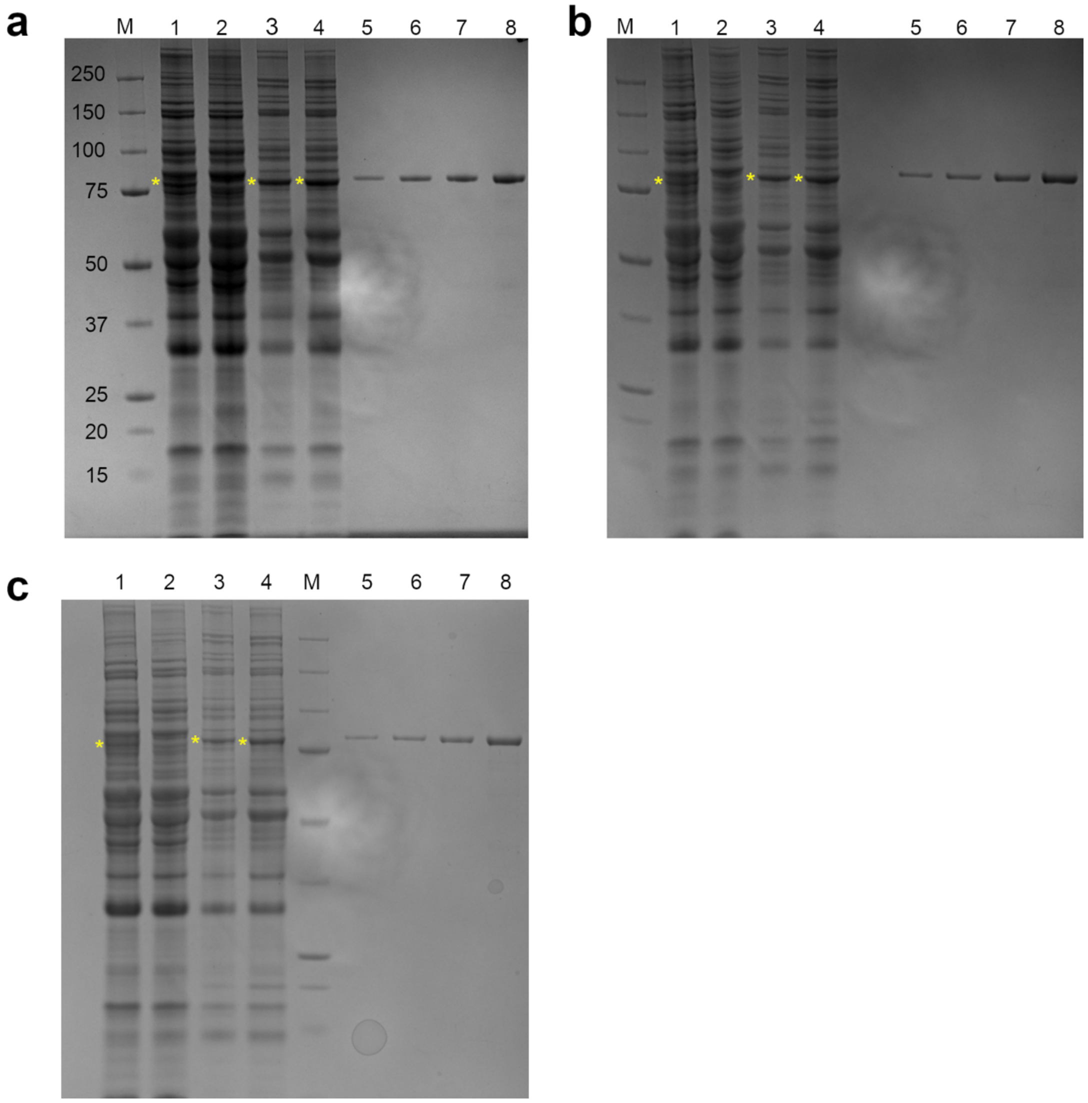

Supplementary Figure 9. Outer-membrane proteins analysis of *Pseudomonas aeruginosa* strains. Proteins of the outer-membrane extracts have been separated by a sodium dodecyl sulfate polyacrylamide gel electrophoresis**.** The experiments were performed three times with three different cultures (**a to c**).

M: molecular weight markers,

1: PAO1 (OD_{600} was 0.964, 0.986 and 1.01 for a, b and c cultures),

2: Δ*pfeA* (OD_{600} was 0.965, 0.902 and 0.926 for a, b and c cultures)*,*

3: Δ*pfeA*(pMMB*pfeA*) (OD_{600} was 0.594, 0.642 and 0.598 for a, b and c cultures),

4: Δ*pfeA*(pMMB*pfeA*R480AQ482A) (OD_{600} was 0.789, 0.764 and 0.720 for a, b and c cultures),

6 to 9: serial dilution of purified PfeA expressed in *E. coli* (47 to 376 ng).

The gel was stained with Coomassie brilliant blue. The bands highlighted by a yellow star were confirmed to be PfeA protein by mass spectrometry. There is more PfeA and PfeAR480A-Q482A protein present in the outer-membrane of Δ*pfeA*(pMMB*pfeA*) and Δ*pfeA*(pMMB*pfeA*R480AQ482A) than PAO1 (and Δ*pfeA* (where there is none); this is despite a higher cell density.

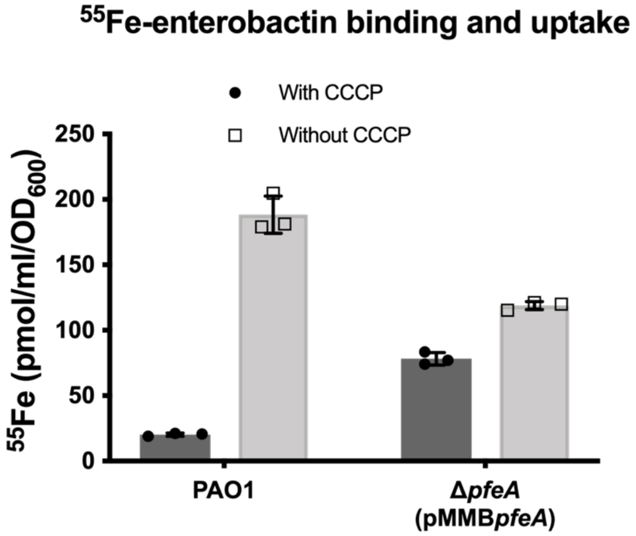

Supplementary Figure 10. ^{55}Fe-enterobactin binding and uptake in PAO1 and Δ*pfeA*(pMMB*pfeA*). Cells were grown and were incubated with or without 200 μM CCCP before initiation of transport assays by the addition of 500 nM ^{55}Fe-enterobactin. After 30 min incubations, the radioactivity accumulated in the bacteria was counted. The results are expressed as pmol of ^{55}Fe-enterobactin bound and transported per ml of cells at an OD_{600} of 1. The experiments have been repeated three times. These data show that the knockout strain with native PfeA is less efficient (although functional) at transport than wild type PAO1.

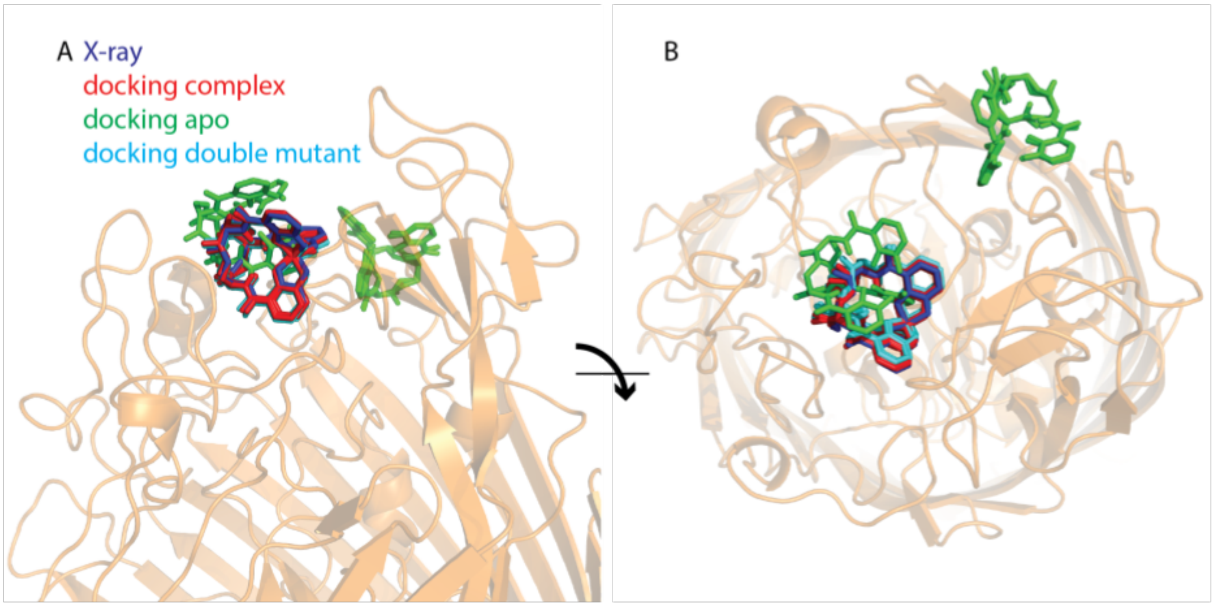

C	Docking complex			Docking apo			Docking double mutant		
Binding free energy (kcal/mol)	-16.9	-16.7	-16.5	-9.9	-9.8	-9.6	-14.9	-14.4	-14.3
Rmsd from X-ray (Å)	0.22	0.46	0.43	20.6	20.8	6.8	0.68	0.65	0.79

Supplementary Figure 11. Fe^{3+}-enterobactin docking in PfeA structures. a-b Docking positions for complex (red), apo (green) and double mutant (cyan) compared to the X-ray (blue). Three most favorable docking positions are shown for each setup. Their overlap with the X-ray position indicates the uniqueness and validity of the proposed binding site. **c** In the table, we reported the binding free energies and distance from the X-ray complex calculated with r.m.s.d. for the three best poses. Closest conformations are highlighted in magenta.

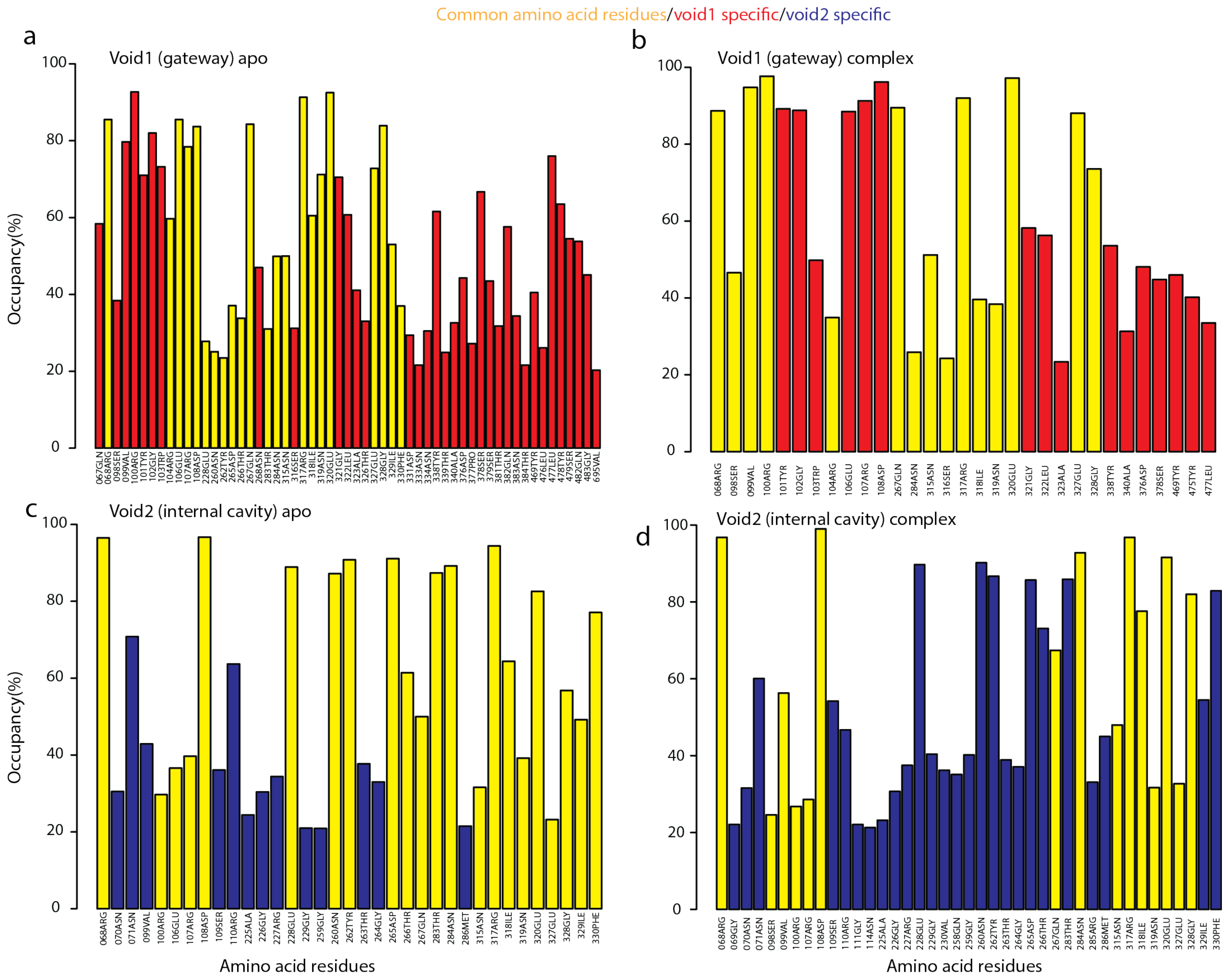

Supplementary Figure 12. **Analysis of the residues forming the voids**. Occupancy of residues forming the void1 (gateway) and the void2 (internal cavity) in apo simulations, (**a**) and (**c**) respectively. Gateway specific residues are shown in red, internal cavity specific residues are shown in blue, while the common residues are shown in yellow. Occupancy of residues forming the void1 (gateway) and the void2 (internal cavity) in the complex simulations, (**b**) and (**d**) respectively. Gateway specific residues are shown in red, internal cavity specific residues are shown in blue, while the common residues are shown in yellow. Compared to the apo (**a-c**) we observe that the number of residues involved in forming internal cavity is significantly larger whilst the number of residues constituting the gateway is smaller. This indicates the effect of the Fe^{3+}-enterobactin binding on the cavities below the binding site.

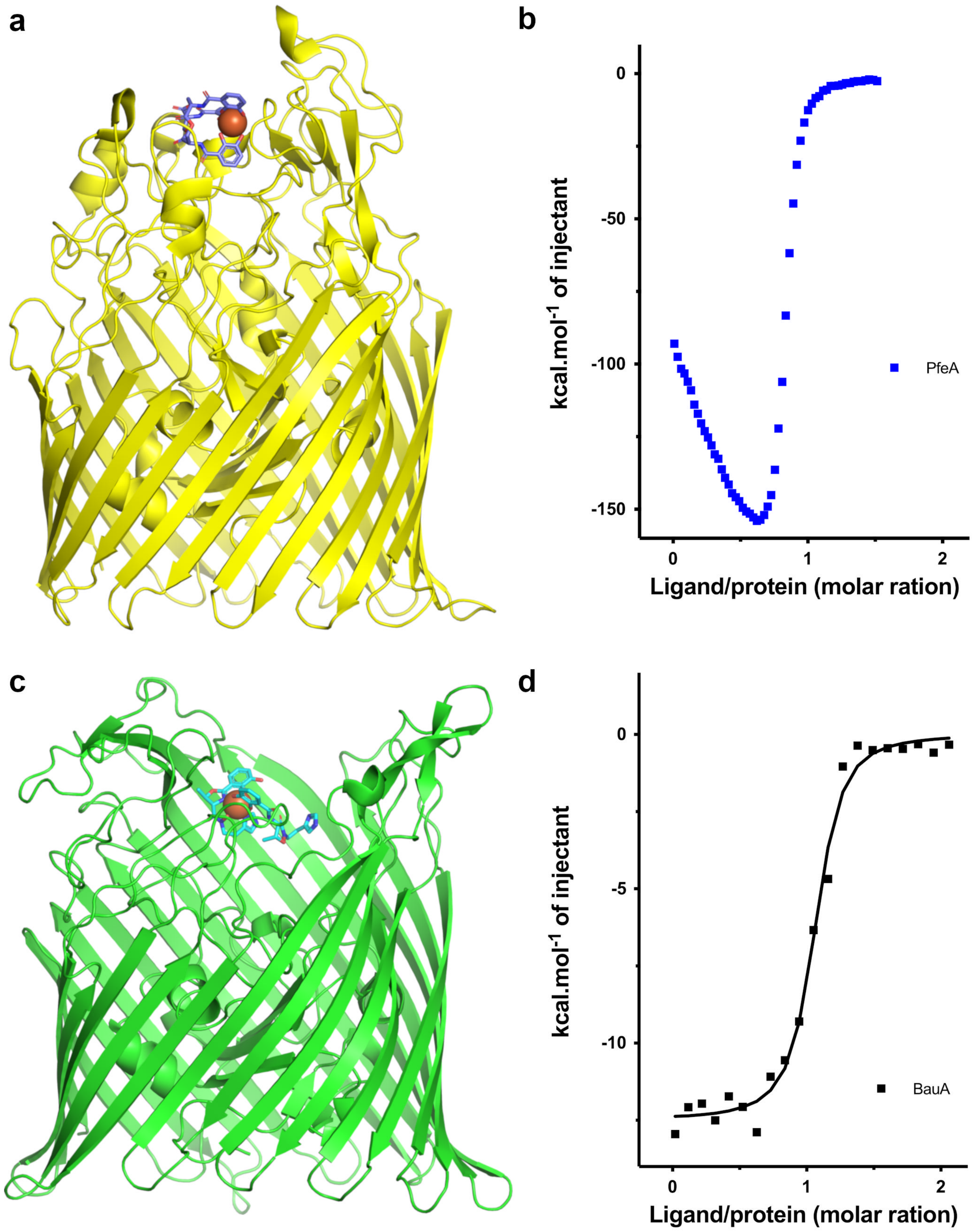

Supplementary Figure 13. PfeA and BauA comparison. PfeA extensive loops, position of the binding site (**a**) and ITC profile (**b**) suggest a two-site binding model. A previous study[8] showed that BauA shorter loops, open conformation, position of the binding site in contact with NL1/NL3 (**c**) and ITC profile **(d)** are consistent with the single binding site model. In **a**, PfeA structure is

shown as a yellow cartoon and enterobactin is shown as sticks with carbon atoms colored in blue, nitrogen in dark blue and oxygen in red. In **b**, BauA structure is shown as green cartoon and preacinetobactin/acinetobactin ligand is shown as sticks with carbon atoms colored in cyan, nitrogen in dark blue and oxygen in red. Fe^{3+} are represented as orange spheres.

Supplementary Methods

PfeA purification. Cells were harvested and resuspended in lysis buffer (50 mM sodium phosphate pH 7.5, 250 mM sodium chloride, 10% (v/v) glycerol, 100 µg.ml^{-1} lysozyme and 20 µg.ml^{-1} Deoxyribonuclease I (Sigma-Aldrich) before been lysed by two passages through a cell disruptor at 30 kpsi (Constant Systems). Cells debris were removed by centrifugation (20 min at 10,000 × *g*) and membrane fractions were isolated by ultracentrifugation (100,000 × *g*; 1 h). The supernatant was discarded, and inner membrane proteins were solubilized in lysis buffer with 1% (w/v) *N*-lauroylsarkosine (Sigma-Aldrich). Outer membrane pellet was isolated by ultracentrifugation (100,000 × *g*; 1 h) and solubilized with 50 mM sodium phosphate pH 8, 250 mM sodium chloride and 7% (v/v) octylpolyoxyethylene (octylPOE) (Bachem). Insoluble material was removed by ultracentrifugation (100,000 × *g*; 1 h). The supernatant was applied to a Histrap nickel-sepharose high-performance column (GE Healthcare) equilibrated with 50 mM sodium phosphate, pH 8, 250 mM sodium chloride, 10 mM imidazole and 1% (v/v) octylPOE and washed with the same buffer containing 15 mM imidazole. Protein was eluted with a linear gradient of imidazole (15 to 150 mM). Fractions containing the protein were dialyzed against 50 mM Tris, pH 8, 250 mM sodium chloride, 10% glycerol, 2 mM mercaptoethanol and 1% (v/v) octylPOE before adding Tobacco etch virus protease to cleave the His_7 tag. The protein was applied to a second Nickel affinity column, followed by a Superdex S200 gel filtration chromatography (GE Healthcare) (10 mM Tris, pH 8, 150 mM NaCl, 0.45% (w/v) C_8E_4 (Bachem). The protein fractions were pooled and concentrated.

Quantitative real-time PCR. Triplicate cultures of mutant strains Δ*pfeA*, Δ*pfeA*(pMMB*pfeA*), Δ*pfeA*(pMMB*pfeA*R480AQ482A) and its parent PAO1 wild-type strain were first grown overnight at 30°C in LB broth before being washed, resuspended and cultivated overnight at 30°C in an iron-deficient CAA medium. Overnight cultures were diluted to 0.1 OD_{600} unit into fresh medium and incubated in the presence of 10 µM enterobactin, with vigorous shaking at 30 °C for 16 h. An aliquot, corresponding to 2.5×10^8 cells, was added to the double volume of RNAprotect Bacteria Reagent (Qiagen). Total RNA was extracted by using RNeasy Mini kit (Qiagen), treated with DNase (RNase-Free DNase Set, Qiagen) and purified with an RNeasy Mini Elute cleanup kit (Qiagen). 1 µg of total RNA was reverse transcribed with High Capacity RNA-to-cDNA Kit as specified by the manufacturer (Applied Biosystems). The amounts of specific cDNA were assessed in a StepOne Plus instrument (Applied Biosystems) by using a Power Sybr Green PCR Master Mix (Applied Biosystems) and corresponding primers (pfeAF 5'-GCCGAGACCAGCGTGAAC-3' and pfeAR 5'- GGCCGGATTCGATCTTGTT-3') with uvrD transcripts as an internal control (uvrDF 5'- CTACGGTAGCGAGACCTACAACAA-3' and uvrDR 5'- GCGGCTGACGGTATTGGA-3'). The transcript levels of pfeA gene in a given strain were normalized with those of uvrD and expressed as a ratio (fold change) to the condition used as a reference (wild-type strain).

Supplementary References

1. Robert X, Gouet P. Deciphering key features in protein structures with the new ENDscript server. *Nucleic Acids Res.* **42**, W320-324 (2014).

2. Wallace AC, Laskowski RA, Thornton JM. LIGPLOT: a program to generate schematic diagrams of protein-ligand interactions. *Protein Eng.* **8**, 127-134 (1995).

3. Cobessi D, Celia H, Pattus F. Crystal structure at high resolution of ferric-pyochelin and its membrane receptor FptA from Pseudomonas aeruginosa. *J. Mol. Biol.* **352**, 893-904 (2005).

4. Cobessi D, Celia H, Folschweiller N, Schalk IJ, Abdallah MA, Pattus F. The crystal structure of the pyoverdine outer membrane receptor FpvA from Pseudomonas aeruginosa at 3.6 angstroms resolution. *J. Mol. Biol.* **347**, 121-134 (2005).

5. Ferguson AD, Chakraborty R, Smith BS, Esser L, van der Helm D, Deisenhofer J. Structural basis of gating by the outer membrane transporter FecA. *Science* **295**, 1715-1719 (2002).

6. Ferguson AD, Hofmann E, Coulton JW, Diederichs K, Welte W. Siderophore-mediated iron transport: crystal structure of FhuA with bound lipopolysaccharide. *Science* **282**, 2215-2220 (1998).

7. Brillet K *et al.* Pyochelin Enantiomers and Their Outer-Membrane Siderophore Transporters in Fluorescent Pseudomonads: Structural Bases for Unique Enantiospecific Recognition. *J. Am. Chem. Soc.* **133**, 16503-16509 (2011).

8. Moynie L *et al.* Preacinetobactin not acinetobactin is essential for iron uptake by the BauA transporter of the pathogen Acinetobacter baumannii. *Elife* **7**, e42270 (2018).
