# Supplementary material for: The complex of ferric-enterobactin with its transporter from Pseudomonas aeruginosa suggests a two-site model
Source: Nat Commun. 2019 Aug 14;10:3673. doi: 10.1038/s41467-019-11508-y (PMC6694100; doi:10.1038/s41467-019-11508-y)
Supplement: Supplementary file 3 — Description of Additional Supplementary Files [file 41467_2019_11508_MOESM3_ESM.pdf]

Description of Additional Supplementary Files

File Name: Supplementary Data 1

Description: Control ITC for PfeA native. (The raw file is PfeANative-control.itc)

File Name: Supplementary Data 2

Description: Enterobactin titration with PfeA native. (The raw file is PfeANative-Entro-titra.itc)

File Name: Supplementary Data 3

Description: Control ITC for PfeAG324V. (The raw file name is PfeAG324V-control.itc)

File Name: Supplementary Data 4

Description: Enterobactin titration with PfeAG324V. (The raw file is PfeAG324V-Entro-titra.itc)

File Name: Supplementary Data 5

Description: Control ITC for PfeAQ482A. (The raw file is PfeAQ482A-control.itc)

File Name: Supplementary Data 6

Description: Enterobactin titration with PfeAQ482A. (The raw file is PfeAQ482A-Entro-titra.itc)

File Name: Supplementary Data 7

Description: Control ITC for PfeAR480A. (The raw file is PfeAR480A-control.itc)

File Name: Supplementary Data 8

Description: Enterobactin titration with PfeAR480A. (The raw file is PfeAR480A-Entro-titra.itc)

File Name: Supplementary Data 9

Description: Control ITC for PfeA for double mutant R480AQ482A. (The raw file is

PfeAR480AQ482A-control.itc)

File Name: Supplementary Data 10

Description: Enterobactin titration with double mutant R480AQ482A. (The raw file PfeAR480AQ482A-Entro-titra.itc)

File Name: Supplementary Data 11

Description: The raw counting device data used to create Figure 6a. (The raw file is Supplementary Data 11.xls)
